# CD56-Negative Extranodal Natural Killer/T-Cell Lymphoma: A Retrospective Study in 443 Patients Treated by Chemotherapy With or Without Asparaginase

**DOI:** 10.3389/fimmu.2022.829366

**Published:** 2022-03-17

**Authors:** Jing Yang, Pengfei Li, Yingshi Piao, Xindi Liu, Liqiang Wei, Wei Sang, Luo Zhang, Liang Wang

**Affiliations:** ^1^ Department of Hematology, Beijing TongRen Hospital, Capital Medical University, Beijing, China; ^2^ Department of Medical Oncology, Sun Yat-sen University Cancer Center, Guangzhou, China; ^3^ Department of Hematology and Oncology, Southern Medical University Cancer Center, Guangzhou, China; ^4^ Hospital of Integrated Traditional Chinese and Western Medicine, Southern Medical University, Guangzhou, China; ^5^ Department of Pathology, Beijing TongRen Hospital, Capital Medical University, Beijing, China; ^6^ Beijing Key Laboratory of Head and Neck Molecular Diagnostic Pathology, Beijing TongRen Hospital, Capital Medical University, Beijing, China; ^7^ Department of Hematology, Affiliated Hospital of Xuzhou Medical University, Xuzhou, China; ^8^ Department of Otolaryngology Head and Neck Surgery, Beijing TongRen Hospital, Capital Medical University, Beijing, China; ^9^ Beijing Key Laboratory of Nasal Disease, Beijing Institute of Otolaryngology, Beijing, China; ^10^ Beijing Advanced Innovation Center for Big Data-Based Precision Medicine, Beihang University & Capital Medical University, Beijing TongRen Hospital, Beijing, China

**Keywords:** extranodal natural killer/T cell lymphoma, tumor microenvironment, prognosis, asparaginase, bioinformatics analysis

## Abstract

**Objective:**

Extranodal natural killer/T cell lymphoma (NKTCL) is an aggressive EBV-related lymphoma, originating from NK cells or T cells. Previous study demonstrated that CD56 negative NKTCL should be recognized as a distinct subtype. In this study, the value of CD56 in NKTCL is validated in the era of asparaginase, and genomic analysis was done to dissect the differences between CD56-negative and positive NKTCL.

**Methods:**

443 patients with newly diagnosed NKTCL were enrolled in this retrospective study, and correlation between CD56 positivity and survival outcomes was analyzed. The gene sequencing data was downloaded (http://www.biosino.org/node/project/detail/OEP000498), and bioinformatics analysis was done to delineate the tumor microenvironment and differentially expressed genes.

**Results:**

CD56 was expressed in 337 patients (76.1%). Within a median follow-up time of 51 months, the 5-year overall survival (OS) and progression free survival (PFS) rates were 63.8% and 51.9%, respectively. For the whole cohort, patients who were CD56-positive had superior OS (5-year OS, 86.2% vs. 51.9%, p=0.019) and PFS (5-year PFS, 55.9% vs. 40.1%, p=0.016). For patients in early stage disease, CD56 positivity was associated with superior OS and PFS (p=0.008 and 0.005, respectively). In patients who received non-asparaginase-based chemotherapy, CD56-negative was associated with shorter OS and PFS (p<0.001), and in patients who received asparaginase-based chemotherapy, CD56-negative was not related to inferior OS and PFS (p=0.093 and p=0.829, respectively). The genomic analysis demonstrated that CD56 positive NKTCL probably originated from NK cells and CD56 negative NKTCL originated from T cells. CD56 positive NKTCL had significantly higher proportion of resting NK cells, activated NK cells, and activated CD8+ and CD4+ T cells in the tumor microenvironment.

**Conclusions:**

CD56 negative NKTCL differs from CD56 positive NKTCL in both the tumor microenvironment and survival outcomes, and asparaginase-based treatment may overcome the poor prognosis brought by CD56 negativity.

## Introduction

Extranodal natural killer/T cell lymphoma (NKTCL), nasal type, is a distinct entity of non-Hodgkin’s lymphoma (NHL) with unique epidemiologic, clinical, histologic and etiologic features. NKTCL is rare in the Western countries, but is more prevalent in East Asia and Latin America (1). Although the standard therapy for NKTCL is not well established, radiotherapy plays a key role in the treatment of early-stage disease (2), and increasing studies have demonstrated the value of systemic chemotherapy in localized disease as reducing the distant relapses (3, 4). Due to the high expression of multi-drug resistance (MDR) gene in NKTCL cells (5), anthracycline-based chemotherapy regimens are no longer recommended in the treatment of NKTCL. Recently, increasing asparaginase-based regimens have proven highly efficacious in the treatment of NKTCL (4, 6–10).

NKTCL is featured by frequent necrosis and angioinvasion. The tumor cells usually express cytoplasmic CD3 and CD56, and have a cytotoxic immunophenotype, expressing perforin, granzyme B (gzm B), and T cell-restricted intracellular antigen (TIA-1) (11). Most cases derive from NK cells that express CD3ϵ and CD56, and lack T-cell receptor (TCR) gene rearrangement, while a very small proportion of cases originate from T cells, with expression of cCD3ϵ and/or sCD3, cytotoxic molecules, and Epstein-Barr virus (EBV) but are negative for CD56 (12–15). Previous studies reported that CD56-negative NKTCL should be regarded as a distinct lymphoma subtype (16, 17). In a large cohort of patients with early-stage NKTCL, CD56-negative NKTCL showed a significantly poorer survival outcome than CD56-positive NKTCL, regardless of the treatment strategies (16). To explore a more effective therapeutic strategy for CD56-negative NKTCL, it is important to examine the response and failure rate of chemotherapy with asparaginase-based regimens in a large number of CD56-negative NKTCL cases.

Hence, we conducted a retrospective study in a large cohort of 443 patients, to identify if patients of CD56-negative NKTCL had a worse outcome than patients of CD56-positive NKTCL, especially who received chemotherapy with or without asparaginase-based regimens. Furthermore, a genomic analysis was done to dissect the differences between CD56-negative and positive NKTCL.

## Materials and Methods

### Patients

Between January 2000 and December 2020, 443 patients with newly diagnosed NKTCL, for whom both detailed pathologic and clinical information were available, were treated in Beijing Tongren Hospital and Sun Yat-sen University Cancer Center, and included in this retrospective analysis. The criteria for case inclusion were as follows: pathologically confirmed diagnosis of NKTCL according to the criteria of the World Health Organization (WHO) classification (15); no previous malignancy; no previous treatment for NKTCL; received at least two cycles of chemotherapy or radiotherapy; adequate pathologic and clinical information. Patients who were negative for EBV-encoded RNA (EBER) were excluded from our study. This study was approved by the Ethic Committee of Beijing Tongren Hospital and Sun Yat-sen University Cancer Center. The need for informed consent was waived because all patients had been de-identified in our datasets.

### Pathological Evaluation

Patient pathological records and original histopathologic slides were reviewed by one lymphoma pathologist (YSP) to confirm the diagnosis again. A standard panel of immunohistochemistry staining, including CD20, cCD3ϵ, CD16, CD56, Ki-67, TIA-1, perforin, granzyme B, and *in situ* hybridization for EBER, was performed. As previously reported (18), CD56-positive NKTCL is defined as expression of NK/T cell antigens, including either CD56+, cCD3ϵ+, and positive for cytotoxic molecules, while CD56-negative NKTCL is defined as cCD3ϵ+ and CD56-, but positive for both cytotoxic molecules and EBER. Staining for CD56 was considered positive when 10% or more of the abnormal lymphoid cells showed positive immunoreactivity.

### Clinical Information

All available clinical information including survival outcomes was collected for analysis. Patients were staged based on the Ann-Arbor staging system (Both MRI and CT scan were more commonly used to stage patients diagnosed before 2010, and PET-CT dominated after 2010, with a total of 320 patients having PET-CT staging).

All patients enrolled in the study received treatment. One of the following initial treatment strategies was delivered: (1) chemotherapy followed by involved field radiotherapy (IFRT); (2) sandwich protocols (IFRT after an initial 2 to 4 cycles of chemotherapy, followed by 2 to 4 cycles of chemotherapy as consolidation); (3) chemotherapy alone; (4) IFRT alone.

The initial chemotherapy regimens included the following: (1) Anthracycline-based regimens (or non-Asparaginase-based regimens) were CHOP (cyclophosphamide, doxorubicin, vincristine, and prednisone) or CHOP-like regimens, and EPOCH (etoposide, doxorubicin, vincristine, cyclophosphamide, and prednisone); (2) Asparaginase-based regimens were CHOP-L (CHOP plus L-asparaginase), EPOCHL (EPOCH plus L-asparaginase), GELOX/P-GEMOX (gemcitabine, oxaliplatin, and L-asparaginase or Pegaspargase), and SVILE (dexamethasone, vindesine, ifosfamide, L-asparaginase, etoposide). After at least 2 cycles of chemotherapy, patients with early stage disease received IFRT at a total dose of 50Gy to 68 Gy. CT scan, MRI scan, or PET/CT scan was performed to evaluate the treatment responses every two courses of chemotherapy (19, 20). After completion of treatment, patients were followed up every three months for the first two years, every six months for the next three years, and yearly thereafter or whenever clinically indicated. Patients who received auto-HSCT or other clinical trials were not censored in our study.

### NKTCL Dataset and Preprocessing

The sequencing data (WGS, WES, and RNA-seq) of NKTCL used in this study can be viewed and downloaded in NODE (http://www.biosino.org/node) using the accession No. OEP000498 (21). The detailed mutation calls, CNV, and RNA-seq expression data can be downloaded from the Mendeley Dataset, accessible through https://data.mendeley.com/datasets/7gwtb7mgrr/draft?a=85eac518-0f19-41f8-aa58-69ed36b66e41. All NKTCL samples were sorted by CD56 expression level from low to high, and they were divided into the high expression group and the low expression group (in our study, CD56 was positive in 76.1% patients of NKTCL, thus, we arbitrarily assigned the top 75% samples as high expression group and the bottom 25% samples as low expression group).

To quantify the proportions of immune cells in the NKTCL samples, we used the CIBERSORT method with the LM22 gene signature from CIBERSORT website, which allows for sensitive and specific discrimination of 22 human immune cell phenotypes, including B cells, T cells, natural killer cells, macrophages, DCs, and myeloid subsets. CIBERSORT is a deconvolution algorithm and it uses a set of reference gene-expression values (a signature with 547 genes) considered a minimal representation for each cell type and, based on these values, infers cell type proportions in data from many kinds of tumor samples or other samples with mixed cell types using support vector regression. The gene expression profile was prepared according to the standard annotation file samples provided by CIBERSORT web portal, and the data were analysed by CIBERSORT.R, which is downloaded from the CIBERSORT web portal (http://cibersort.stanford.edu/), with the algorithm run using the LM22 signature file and 1000 permutations. Proportions of stromal cells were estimated by applying the Microenvironment Cell Populations-counter method, which allows for robust quantification of the absolute abundance of eight immune and two stromal cell populations in heterogeneous tissues from transcriptomic data.

To identify different genes associated with the expression of CD56, we grouped all patients into groups based on the expression of CD56. Differentially expressed genes among these groups were determined using the R package limma, which implements an empirical Bayesian approach to estimate gene-expression changes using moderated t tests. The differentially expressed genes among groups were determined by significance criteria (abs(log2FC)>1 and adjusted P value<0.05) as implemented in the R package limma. The heatmap was drawn by pheatmap R package according to the differentially expressed genes. Then we performed unsupervised analyzing and hierarchical clustering of common differentially expressed genes based on expression data.

The signature genes were annotated and enriched by clusterProfiler R package. Then in order to have further insight into the function of genes and their interactions, we used the pathway enrichment analysis of DEGs to those signature genes. ClusterProfiler R package was applied to the analysis of KEGG pathways to present the top 10 pathways.

### Statistical Analysis

Treatment responses were evaluated as previously reported (19, 20). Due to the relatively large time span of our study, two different criteria were adopted. For patients who used CT and MRI for response evaluation, the criteria reported by Cheson et al. in 1999 was used (20). For patients who used PET-CT for response evaluation, the criteria reported by Cheson et al. in 2007 was used (19). Overall survival (OS) was calculated from the date of diagnosis to the date of death or the last date of follow-up visit, whichever came first. Progression-free survival (PFS) was measured from the date of diagnosis to the date of confirmed disease progression, relapse, death, or the date of the last follow-up visit, whichever came first. The correlation between CD56 expression and clinicopathologic features of the NKTCL patients was evaluated by the chi-squared test. The Kaplan-Meier product-limit method was used to estimate the probability of OS and PFS, and survival curves were compared for statistical differences by using the log-rank test. Both univariate and multivariate analyses were done using the forward selection method in the stepwise Cox proportional hazard model. Two-sided P values less than 0.05 was considered statistically significant. All statistical analysis was conducted using SPSS software (SPSS Standard Version 20.0, SPSS Inc., Chicago, IL, USA).

## Results

### Patient Characteristics

A total of 443 patients fulfilled the inclusion criteria, and the clinical characteristics are presented in **Table 1**. The median age was 44 years (range 18–79). Three hundred and forty-nine patients (78.8%) had a good performance status (Eastern Cooperative Oncology Group 0-1). The majority of patients initially presented with early stage disease (n=389, 87.7%) or upper aerodigestive tract (UAT) tumors (n=309, 69.8%). 75.6% of patients were categorized as low risk according to international prognostic index (IPI), and 60.3% were in group 1 or 2 according to Korean prognostic index (KPI).

**Table 1:** Clinical characteristics and CD56 expression status of 443 patients at diagnosis with NKTCL.

Characteristics	All cases (n=443,100%)	CD56-positive Group (n=337, 76.1%)	CD56-negative Group (n=106, 23.9%)	*P* value
Gender				0.230
Male	299 (67.5%)	233 (69.1%)	66 (62.3%)	
Female	144 (32.5%)	104 (30.9%)	40 (37.7%)	
Age at diagnosis >60 (years)	64 (14.4%)	44 (13.1%)	20 (18.9%)	0.185
ECOG PS score ≥2	94 (21.2%)	61 (18.1%)	33 (31.1%)	0.006*
Subtypes				0.974
Nasal cavity	309 (69.8%)	236 (70.0%)	73 (68.9%)	
Extranasal UAT	93 (21.0%)	70 (20.8%)	23 (21.7%)	
Extra UAT	41 (9.2%)	31 (9.2%)	10 (9.4%)	
B-symptoms	205 (46.3%)	148 (43.9%)	57 (53.8%)	0.096
Primary tumor invasion	268 (60.5%)	198 (58.8%)	70 (66.0%)	0.221
Extranodal sites ≥ 2	87 (19.6%)	67 (19.9%)	20 (18.9%)	0.929
Regional lymphadenopathy	191 (43.1%)	143 (42.4%)	48 (45.3%)	0.686
Elevated serum LDH level	120 (27.1%)	92 (27.3%)	28 (26.4%)	0.957
Ann Arbor Stage				0.886
I, II	389 (87.8%)	295 (87.5%)	94 (88.7%)	
III, IV	54 (12.2%)	42 (12.5%)	12 (11.3%)	
IPI score				0.047*
0, 1	335 (75.6%)	263 (78.0%)	72 (67.9%)	
≥ 2	108 (24.4%)	74 (22.0%)	34 (32.1%)	
KPI score				0.318
0, 1	267 (60.5%)	208 (61.7%)	59 (55.7%)	
≥ 2	176 (39.5%)	129 (38.3%)	47 (44.3%)	

ECOG PS, Eastern Cooperative Oncology Group performance status; Extranasal UAT, Extranasal upper aerodigestive tract; Extra UAT, extraupper aerodigestive tract; LDH, lactate dehydrogenase; IPI, International Prognostic Index; KPI, Korean Prognostic Index.

*Indicates statistically significant.

### Association of CD56 With Pathologic and Clinical Characteristics

**Figure 1:**
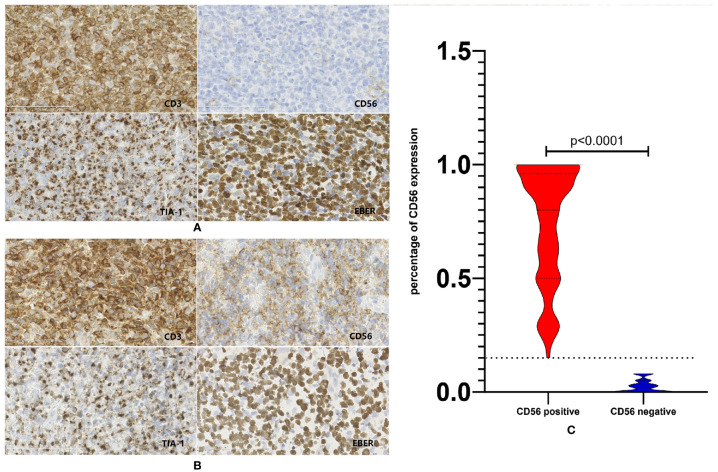
Expression status of CD56 in patients with NKTCL. **(A)** Representative case of NKTCL defined as CD56 negative. **(B)** Representative case of NKTCL defined as CD56 positive. **(C)** Violin plot of CD56 expression status in the whole cohort of NKTCL.

**Figures 1A, B** represented the typical CD56 negative and positive NKTCL cases. As is shown in **Figure 1C**, about half patients with CD56 positive NKTCL had CD56 expression in more than 80% of lymphoma cells. Categorical associations between CD56 expression and main clinical features are summarized in **Table 1**. CD56 was expressed in 337 patients (76.1%). The CD56-negative group showed statistically significant increase in cases with poor ECOG score and IPI score compared with the CD56-positive group (p=0.006 and p=0.047, respectively). No categorical association was observed with other clinical features, such as gender, age, primary tumor invasion, number of extranodal sites, serum LDH level, and Ann-Arbor stage.

### Treatment Modalities and Responses

The initial treatment modalities were as follows: (1) for early stage disease, 293 (75.3%) patients received chemotherapy followed by radiotherapy, 80 (20.6%) patients received chemotherapy alone, and 11 patients received radiotherapy alone; Two hundred and nineteen (57.9%) patients received asparaginase-based chemotherapy and the other 159 (42.1%) patients received non-asparaginase-based chemotherapy. (2) For advanced stage disease, 54 (100%) patients received chemotherapy, and eight (14.8%) patients with local residual disease received radiotherapy after induction chemotherapy; Twenty-six (48.1%) patients received asparaginase-based chemotherapy and the other 28 (51.9%) patients received non-asparaginase-based chemotherapy. No significant difference was found in the treatment modalities as well as chemotherapy regimens between patients with different CD56 expression status (p=0.543 and p=0.113, respectively). The treatment responses were evaluated in 422 (95.2%) patients. 304 (72.0%) patients and 68 (16.1%) patients achieved complete response (CR) and partial response (PR), respectively, thus the overall response rate (ORR) was 88.1%. The CR rate was significantly higher in patients who were treated with asparaginase-based therapy (75.8 vs. 64.1%, p = 0.006). ORR of patients with CD56-positive and CD56-negative expression were 89.3% and 82.1%, respectively (p = 0.609).

### Prognostic Role of CD56 in Patients With NKTCL

**Figure 2:**
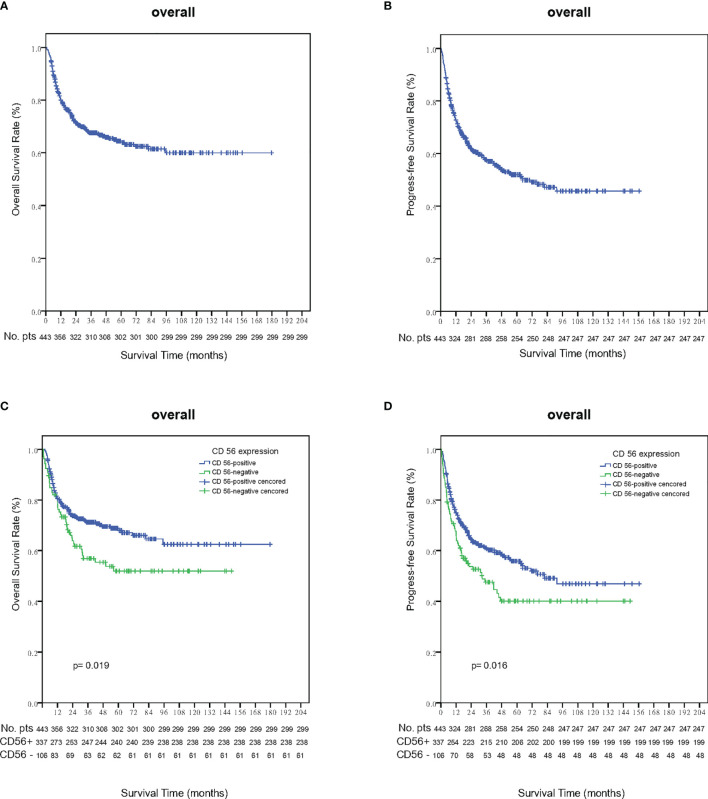
Survival outcomes in the whole cohort of NKTCL. **(A)** Overall survival (OS); **(B)** Progression-free survival (PFS); **(C)** Overall survival (OS) analysis according to the expression status of CD56 in the whole cohort of NKTCL; **(D)** Progression-free survival (PFS) analysis according to the expression status of CD56 in the whole cohort of NKTCL.

**Figure 3:**
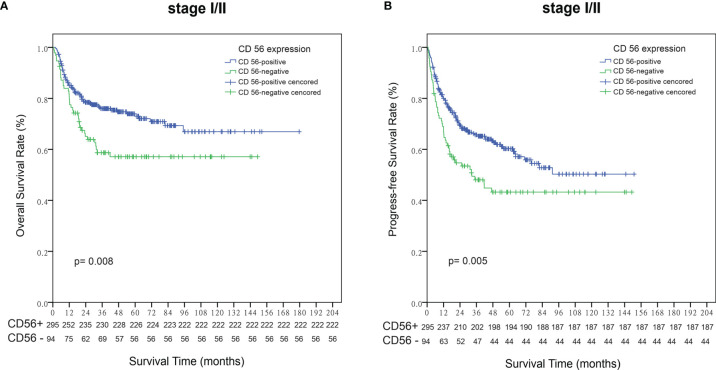
Survival analysis according to the expression status of CD56 in patients with early stage NKTCL. **(A)** Overall survival (OS); **(B)** Progression-free survival (PFS).

**Figure 4:**
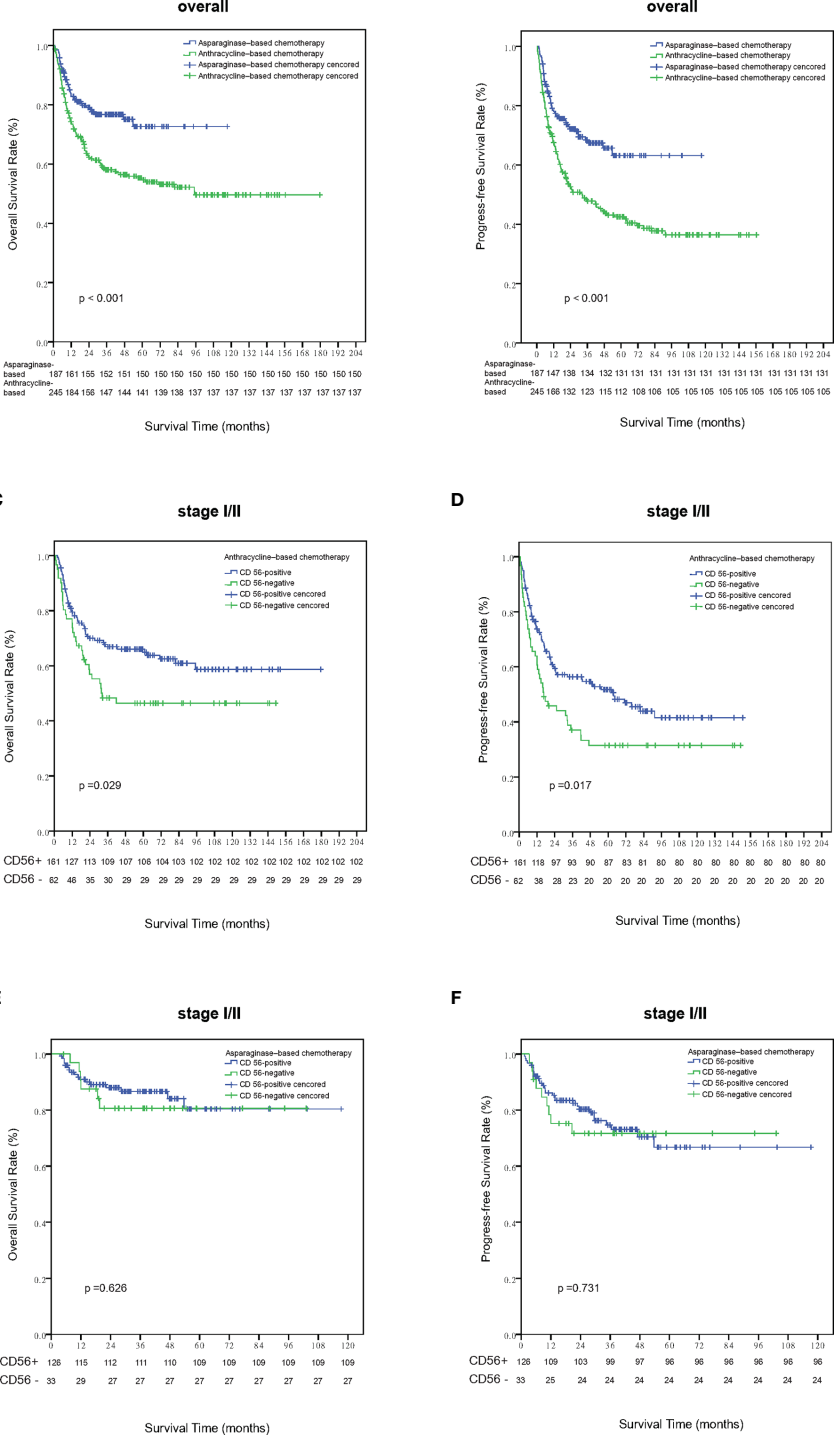
Survival analysis according to different treatment strategies. Patients treated with asparaginase-based chemotherapy had significantly better overall survival **(A)** and progression-free survival **(B)** than those treated with anthracycline-based regimens. Out of patients with stage I-II disease who were treated with anthracyline-based chemotherapy, patients who were CD56 negative had significantly inferior overall survival **(C)** and progression-free survival **(D)** than those CD56 positive ones. Out of patients with stage I-II disease who were treated with asparaginase–based chemotherapy, the expression status of CD56 was not related with overall survival **(E)** and progression-free survival **(F)**.

Within a median follow-up time of 51 months (range, 1 to 180 months), 196 patients experienced disease progression or relapse and 145 patients died. The 5-year OS and PFS rates for all 443 patients were 63.8% (95% CI, 58.7–68.9%) and 51.9% (95% CI, 46.6–57.2%), respectively (**Figures 2A, B**). Patients who were CD56-positive had significantly superior OS (5-year OS, 86.2% vs. 51.9%, p = 0.019, **Figure 2C**) and PFS (5-year PFS, 55.9% vs. 40.1%, p =0.016, **Figure 2D**). For patients in the early stage disease, CD56 positivity was associated with superior OS and PFS (p=0.008, **Figure 3A** and p=0.005, **Figure 3B**, respectively). However, for patients in the advanced stage disease, CD56 positivity was not related to OS and PFS (p=0.857 and p=0.856, respectively. **Supplementary Figures 1A, B**). In patients who received non-asparaginase-based chemotherapy, CD56-negative was associated with inferior OS and PFS (both p<0.001), while in patients who received asparaginase-based chemotherapy, CD56-negative was not related to OS and PFS (p=0.093 and p=0.829, respectively). Asparaginase-based chemotherapy significantly improved the prognosis compared with non-asparaginase-based regimens in the whole cohort (5-year OS, 75.0% vs. 55.0%, p<0.001, **Figure 4A**; 5-year PFS, 63.2% vs. 42.5%, p<0.001, **Figure 4B**), and in patients with early stage disease (5-year OS, 80.6% vs. 59.5%, p<0.001; 5-year PFS, 68.2% vs. 45.8%, p<0.001). Subgroup analysis showed that CD56-negative was associated with inferior OS and PFS (p=0.029, **Figure 4C** and p=0.017, **Figure 4D**, respectively) in patients with early stage disease who received non-asparaginase-based chemotherapy, but not related to OS and PFS (p=0.626, **Figure 4E** and p=0.731, **Figure 4F**, respectively) in early stage patients treated with asparaginase-based regimens. Compared with non-asparaginase-based chemotherapy, asparaginase-based chemotherapy obviously improved OS and PFS (5-year OS, 80.6% vs. 46.4%, p<0.001 and 5-year PFS, 66.8% vs. 31.4%, p<0.001) for patients with CD56-negative early stage disease (**Table 2**).

**Table 2:** Treatment outcomes of patients with CD56-positive vs. CD56-negative NKTCL.

Characteristics		5-year OS (%)			5-year PFS (%)		
		CD56-positive group (%) (n=337)	CD56-negative group (%) (n=106)	P value	CD56-positive group (%) (n=337)	CD56-negative group (%) (n=106)	P value
Gender	Male	64.6	45.8	0.017*	54.9	35.4	0.002*
	Female	75.0	61.4	0.314	57.8	47.6	0.867
Age at diagnosis (years)	≤60	69.1	60.7	0.364	56.6	45.5	0.245
	>60	61.3	15.9	0.004*	50.2	16.2	0.002*
ECOG PS score	0,1	72.7	55.3	0.012*	59.6	46.3	0.052
	≥2	46.2	45.1	0.649	38.9	27.5	0.081
Subtypes	Nasal cavity	71.7	61.2	0.178	58.2	49.5	0.191
	Extranasal UAT	68.5	40.3	0.030*	56.3	20.1	0.009*
	Extra UAT	41.0	0.0	0.337	34.4	0.0	0.574
B-symptoms	Prestent	65.7	39.5	0.018*	54.4	30.7	0.017*
	Absent	69.7	67.3	0.652	57.0	51.4	0.463
Serum LDH level	Elevated	53.8	37.1	0.247	43.7	24.6	0.064
	Normal	73.2	57.3	0.034*	60.4	45.8	0.080
Ann Arbor Stage	I, II	73.0	57.1	0.008*	60.2	43.2	0.005*
	III, IV	30.8	20.0	0.857	25.1	21.9	0.856
IPI score	0,1	74.6	60.4	0.015*	62.5	47.2	0.028*
	≥2	44.1	32.2	0.644	32.4	24.2	0.918
KPI score	0,1	76.6	53.4	0.079	65.0	52.1	0.143
	≥2	54.0	36.6	0.250	40.9	24.5	0.112
Treatment	Chemotherapy	42.2	33,6	0.601	28.8	22.1	0.402
	Chemotherapy and radiotherapy	75.9	61.9	0.108	62.6	49.9	0.175
Chemotherapy regimens	Asparaginase-based	74.3	77.2	0.963	61.2	70.5	0.829
	Non-asparaginase-based	60.6	41.6	0.027*	48.4	28.3	0.017*

ECOG PS, Eastern Cooperative Oncology Group performance status; Extranasal UAT, Extranasal upper aerodigestive tract; Extra UAT, extraupper aerodigestive tract; LDH, lactate dehydrogenase; IPI, International Prognostic Index; KPI, Korean Prognostic Index.

*Indicates statistically significant.

As is shown in **Table 2**, univariate analysis revealed that age, ECOG PS, Subtype, B symptoms, primary tumor invasion, number of extranodal sites, regional lymphadenopathy invasion, LDH level, Ann-Arbor stage, expression status of CD56, IPI score and KPI score correlated significantly with both OS and PFS. Clinical factors that were statistically significant predictors of OS and PFS were included in the multivariate analysis (see **Table 3**), which showed that age, ECOG PS, primary tumor invasion and Ann-Arbor stage were independent prognostic factors for both OS and PFS. KPI score was also an independent prognostic factor for PFS, but failed to be prognostic for OS. The expression status of CD56 had a trend towards independent prognostic factors for OS and PFS (p=0.188 and 0.146, respectively). Next, we performed both univariate and multivariate analysis in patients with early stage disease. As is shown in **Table 4**, age, ECOG PS, primary tumor invasion, regional lymphadenopathy, and CD56 expression status were found to be independent prognostic factors for PFS.

**Table 3:** Results of univariate and multivariate analyses of prognostic factors for OS and PFS in all patients.

Parameter	OS			PFS		
	Univariate analysis	Multivariate analysis		Univariate analysis	Multivariate analysis	
	*P*-value	HR (95% CI)	*P*-value	*P*-value	HR (95% CI)	*P*-value
Age > 60 years	< 0.0001*	2.976(1.876–4.717)	< 0.0001*	0.020*	1.776(1.166-2.710)	0.008*
Gender, male	0.055			0.546		
ECOG PS ≥ 2	< 0.0001*	2.169(1.429-3.289)	< 0.0001*	< 0.0001*	1.770(1.232-2.545)	0.002*
Subtype, Extra UAT	< 0.0001*			< 0.0001*		
B symptoms	0.013*			0.012*		
Primary tumor invasion	< 0.0001*	1.761(1.167-2.653)	0.007*	< 0.0001*	1.595(1.134-2.242)	0.007*
Extranodal sites ≥ 2	0.001*			0.008*		
Regional lymphadenopathy	0.002*			< 0.0001*		
Elevated serum LDH	< 0.0001*			< 0.0001*		
CD 56- negative	0.019*	1.285 (0.885–1.866)	0.188	0.016*	1.269 (0.920–1.751)	0.146
Stage III, IV	< 0.0001*	2.688 (1.447–4.975)	0.002*	< 0.0001*	1.757 (1.002–3.086)	0.049*
IPI score ≥ 2	< 0.0001*			< 0.0001*		
KPI score ≥ 2	< 0.0001*			< 0.0001*	1.767(1.109-2.817)	0.017*

OS, overall survival; PFS, progression-free survival; RR, relative risk; CI, confidence interval; ECOG PS, Eastern Cooperative Oncology Group performance status; Extra UAT, extraupper aerodigestive tract; LDH, lactate dehydrogenase; IPI, International Prognostic Index; KPI, Korean Prognostic Index.

*Indicates statistically significant.

**Table 4:** Results of univariate and multivariate analyses of prognostic factors for OS and PFS in patients with Stage I/II.

Parameter	OS			PFS		
	Univariate analysis	Multivariate analysis		Univariate analysis	Multivariate analysis	
	*P*-value	RR (95% CI)	*P*-value	*P*-value	RR (95% CI)	*P*-value
Age > 60 years	< 0.0001*	2.604(1.675–4.065)	< 0.000*	0.005*	1.812(1.217-2.695)	0.003*
Gender, male	0.024*			0.491		
ECOG PS ≥ 2	< 0.0001*	1.672(1.092-2.564)	0.018*	< 0.0001*	1.730(1.200-2.494)	0.003*
Subtype, Extra UAT	0.471			0.292		
B symptoms	0.146			0.341		
Priamry tumor invasion	< 0.0001*	1.761(1.167-2.653)	0.002*	< 0.0001*	1.595(1.134-2.242)	0.001*
Extranodal sites ≥ 2	0.681			0.823		
Regional lymphadenopathy	0.042*	1.437(0.953-2.165)	0.083	0.002*	1.543(1.092-2.183)	0.014*
Elevated serum LDH	0.038*			0.026*		
CD 56- negative	0.008*	1.451(0.975–2.160)	0.067	0.005*	1.786 (1.250–2.551)	0.024*

OS, overall survival; PFS, progression-free survival; RR, relative risk; CI, confidence interval; ECOG PS, Eastern Cooperative Oncology Group performance status; Extra UAT, extraupper aerodigestive tract; LDH, lactate dehydrogenase; IPI, International Prognostic Index; KPI, Korean Prognostic Index.

*Indicates statistically significant.

### Bioinformatic Analysis Favored CD56 Negative NKTCL to Be a Distinct Entity

**Figure 5:**
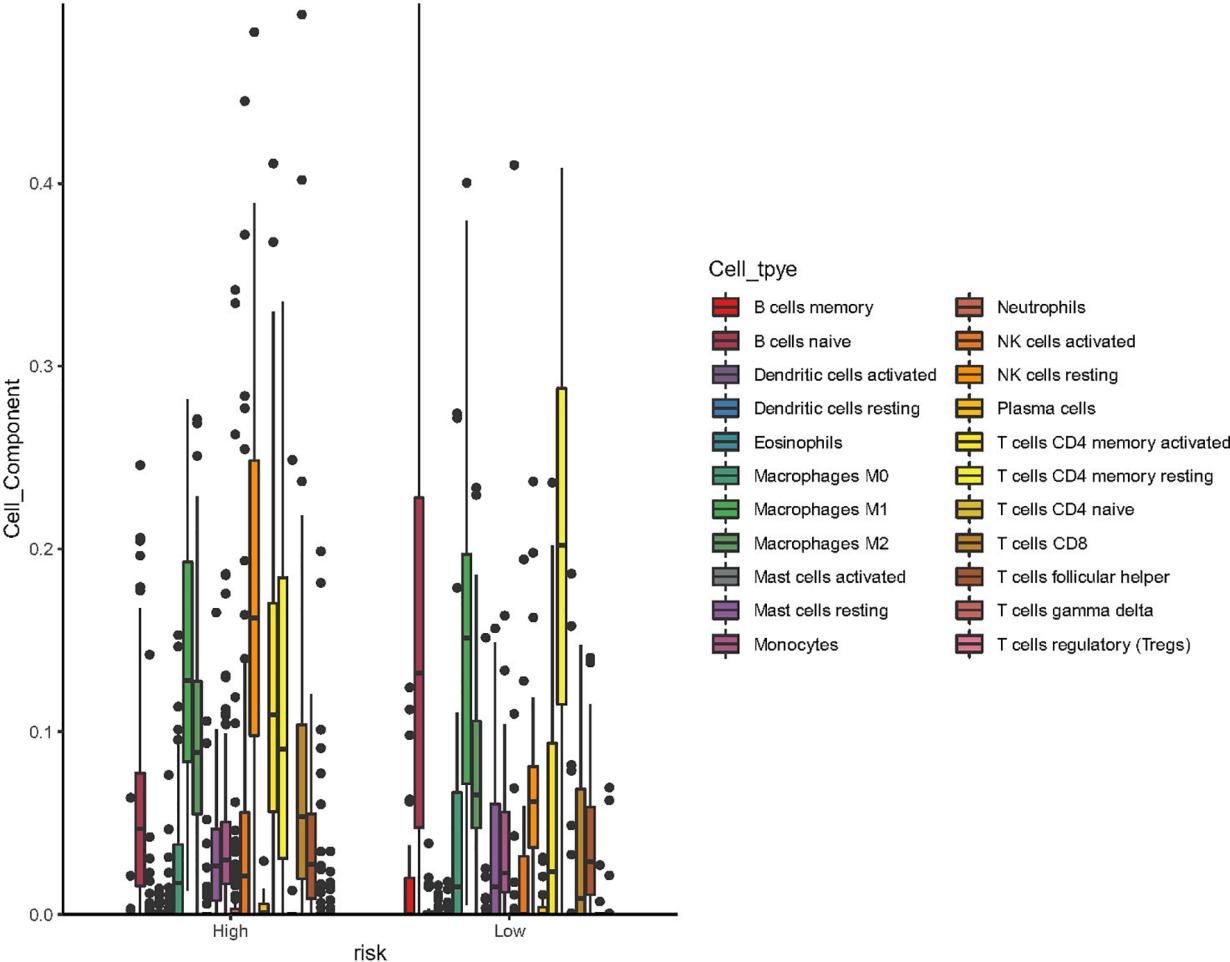
Box plot of the processed data according to their CIBERSORT results (using ggboxplot from ggpubr R package). The figure showed that resting NK cells, activated NK cells, CD8^+^ T cells and activated CD4^+^ T memory cells was significantly higher in patients with high expression of CD56.

**Figure 6:**
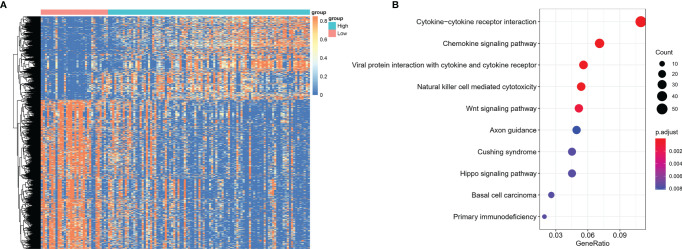
Differentially expressed genes between NKTCL patients with high and low expression of CD56. **(A)** Heatmap shows the differentially expressed genes among groups, which were determined by significance criteria (abs(log2FC)>1 and adjusted P value<0.05) as implemented in the R package limma. The heatmap was drawn by pheatmap R package according to the differentially expressed genes. Unsupervised analyzing and hierarchical clustering of common differentially expressed genes based on expression data. **(B)** Gene annotation enrichment analysis using the clusterProfiler R package was performed on signature genes. The top five pathways with the greatest enrichment were Cytokine-cytokine receptor interaction, Chemokine signaling pathway, Viral protein interaction with cytokine and cytokine receptor, Natural killer cell mediated cytotoxicity, Wnt signaling pathway.

Using CIBERSORT algorithm and the LM22 gene signature, we can evaluate the proportion of various immune cells in the tumor microenvironment of NKTCL. As is shown in **Figure 5**, both resting and activated NK cells, CD8^+^ T cells and activated CD4^+^ memory T cells were significantly elevated in high CD56 expression group. As the heatmap shows in **Figure 6A**, numerous genes were differentially expressed between CD56 high and low expression group. According to the KEGG analysis shown in **Figure 6B**, those differentially expressed genes mainly involve in Cytokine-cytokine receptor interaction(map04060), Chemokine signaling pathway(map04062), Viral protein interaction with cytokine and cytokine receptor(map04061), Natural killer cell mediated cytotoxicity(map04650), and Wnt signaling pathway(map04310).

## Discussion

Most cases of NKTCL are derived from NK cells, which are generally featured by CD56+, surface CD3−, and cytoplasmicCD3ϵ+ (14).However, NKTCL originated from T cells can be less commonly seen, usually with TCR rearrangement (12, 22, 23). CD56 expression is not specific for NKTCL and the diagnosis of NKTCL can still be confirmed for patients with typical morphology and expression of CD3ϵ, cytotoxic proteins and EBER (15). A previous study showed that patients with CD56-negative NKTCL had significantly dismal outcomes, indicating a distinct entity of NKTCL (16).

In the present study, 443 patients with NKTCL were assessed for the expression of CD56, with 76% positivity. Our findings were consistent with previous studies, in which the percentage of CD56 expression ranged from 74-97% (16–18). Our data demonstrated a notable difference in several clinical behaviors between the CD56-positive and CD56-negative groups. There were more patients with poor ECOG PS and higher IPI score in CD56-negative group. Although CD56 expression status did not influence the responses to initial treatment modalities as ORR did not differ between CD56 positive and negative groups, it was found to be a prognostic factor for PFS and OS in both the whole cohort and patients with early stage disease. Meanwhile, CD56 expression status was demonstrated to be an independent prognostic factor for PFS in patients with early stage disease, but failed to show an independent role for predicting OS in those patients. Our findings were consistent with a previous retrospective study, which identified CD56-negative expression was an independent adverse prognostic factor for PFS in early stage disease (16). Only 12 patients with advanced stage disease were CD56 negative, which may explain the failure to reveal independent role for predicting survival outcomes in patients with stage III/IV disease. Moreover, diverse salvage treatment regimens may impact the true value of CD56 expression status in predicting OS.

Currently, combined chemotherapy and radiotherapy is recommended for early-stage NKTCL (2, 6, 7, 24). Anthracycline-based chemotherapy regimens (e.g., CHOP or CHOP-like) were proven unfit for NKTCL, partially due to overexpression of MDR in NKTCL cells (5). Asparaginase-containing regimens have been recognized as standard of care treatment for both early and advanced disease (6, 7, 24–26). Our study showed that CD56-negative expression resulted in reduced PFS and OS rate of early-stage patients who received non-asparaginase-based chemotherapy, while this phenomena was not seen in patients who received asparaginase-based chemotherapy. These findings suggest that asparaginase-based treatment strategies might overcome the poor prognosis of CD56-negative NKTCL.

Xiong et al. successfully proposed the molecular subtypes of NKTCL using multi-omics analysis (21). In their cohort, 24 out of 99 patients had TCR rearrangement, who were defined as true T cells origin. Using a novel algorithm developed by Xiong et al. (based on quantitative gene expression metrics of NK-cell and T-cell associated genes), they divided 102 patients into NK-cell origin (n = 53) and T-cell origin (n = 49). The discrepancy between this novel algorithm and TCR results have not been fully understood (21). However, according to the findings reported by Xiong et al, the genes used for construction of this algorithm were not robustly specific for either T cell or NK cell. Thus, this algorithm needs to be validated further.

In our study, we arbitrarily defined patients with the top 75% expression level of CD56 as high expression group and the bottom 25% as low expression group to explore the differences between different CD56 expression status. As the heatmap shows in **Figure 5**, there was a distinct gene expression mode, indicating a possible different cell origin. The KEGG analysis showed that differentially expressed genes mainly involved in Natural killer cell mediated cytotoxicity and so on, which inferred that NKTCL with high CD56 expression may originate from NK cells. This conclusion was confirmed in the tumor microenvironment analysis of NKTCL, which showed that patients with high CD56 expression had significantly higher proportion of both resting and activated NK cells. Moreover, the activated CD8^+^ and CD4^+^ T cells were significantly higher in patients with high CD56 expression, and these cells could promote the immune attack towards tumors cells and result in good prognosis, which was confirmed by our survival analysis. Intriguingly, resting NK cells dominated in tumor microenvironment of NKTCL with high expression of CD56. Our results were derived from CIBERSORT algorithm, which reflects both the tumoral cells and tumor microenvironment cells. According to the algorithm Xiong et al. conceived to categorize cell origin (21), we reproduced their findings (Please see **Supplementary Figure 2**). Firstly, we compared the RNA level of CD56 in different cell origins, and found that CD56 was significantly elevated in NK-cell origin samples (Please see **Supplemental Figure 3**, p=3.2×10^-6^). Subsequently, we compared the tumor microenvironment status between patients with NK-cell origin and T-cell origin (Please see **Supplemental Figure 4**) using CIBERSORT algorithm. In consistence with our findings, resting NK cells dominated in NK-cell origin group, and we can see the proportion of activated NK cells were much higher in patients with NK-cell origin. Thus, although we know that tumoral NK cells are more likely to be activated ones, the CIBERSORT algorithm has limitations to reflect the whole picture of tumor microenvironment accurately, and maybe sing-cell RNA sequencing data could help better define the NK cells status.

In conclusion, we found that CD56-negative NKTCL significantly correlated with poor outcomes and can further discriminate the prognosis of patients with early stage. The use of asparaginase-based regimens could overcome the dismal impact brought by CD56-negative NKTCL. Moreover, CD56 expression status may infer the cell origin of NKTCL. However, this is a retrospective study, and these results need to be validated in prospective trials.

## Data Availability Statement

The analyzed data sets generated during the study are available from the corresponding author, on reasonable request. The sequencing data (WGS, WES, and RNA-seq) of NKTCL used in this study can be viewed and downloaded in NODE (http://www.biosino.org/node) using the accession No. OEP000498 through the URL: http://www.biosino.org/node/project/detail/OEP000498. The detailed mutation calls, CNV, and RNA-seq expression data can be downloaded from the Mendeley Dataset, accessible through the following link: https://data.mendeley.com/datasets/7gwtb7mgrr/draft?a=85eac518-0f19-41f8-aa58-69ed36b66e41.

## Ethics Statement

This study was approved by the Ethic Committee of Beijing Tongren Hospital and Sun Yat-sen University Cancer Center. The need for informed consent was waived because all patients had been de-identified in our datasets. Written informed consent for participation was not required for this study in accordance with the national legislation and the institutional requirements.

## Author Contributions

LW, LZ, and WS designed this study. JY and PL collected all clinical data and analyzed the data. YP reviewed the pathology reports and evaluated the expression of CD56. XL did the bioinformatics analysis. LQW, PL, WS, LZ, and LW contributed to patient selection and treatment. LW, JY, WS, and PL wrote the paper. All authors read and approved the submission of this paper.

## Funding

This work was supported by grants from the National Natural Science Foundation of China (grant No. 81873450 and 82170181), the Open Research Fund from Beijing Advanced Innovation Center for Big Data-Based Precision Medicine, Beijing Tongren Hospital, Beihang University, and Capital Medical University (grant No. BHTR-KFJJ-202009), and Beijing Hospitals Authority Youth Programme (code: QML20200201) to LW.

## Conflict of Interest

The authors declare that the research was conducted in the absence of any commercial or financial relationships that could be construed as a potential conflict of interest.

## Publisher’s Note

All claims expressed in this article are solely those of the authors and do not necessarily represent those of their affiliated organizations, or those of the publisher, the editors and the reviewers. Any product that may be evaluated in this article, or claim that may be made by its manufacturer, is not guaranteed or endorsed by the publisher.

## Supplementary Material

Supplementary Figure 1 Survival analysis according to the expression status of CD56 in patients with advanced stage NKTCL. **(A)** Overall survival (OS); **(B)** Progression-free survival (PFS).

Supplementary Figure 2 According to the algorithm conceived by Xiong et al. (reported in Cancer Cell. 2020), we reproduced the categorization of NKTCL according to NK-cell or T-cell origin.

Supplementary Figure 3 Using data reported by Xiong et al. (reported in Cancer Cell. 2020), CD56 was significantly elevated in patients with NK-cell origin.

Supplementary Figure 4 CIBERSORT analysis in groups with different cell origins, which were categorized using the algorithm conceived by Xiong et al. (reported in Cancer Cell. 2020).
