# Supplementary figures and images for: CD56-Negative Extranodal Natural Killer/T-Cell Lymphoma: A Retrospective Study in 443 Patients Treated by Chemotherapy With or Without Asparaginase
Source: Front Immunol. 2022 Mar 17;13:829366. doi: 10.3389/fimmu.2022.829366 (PMC8968031; doi:10.3389/fimmu.2022.829366)

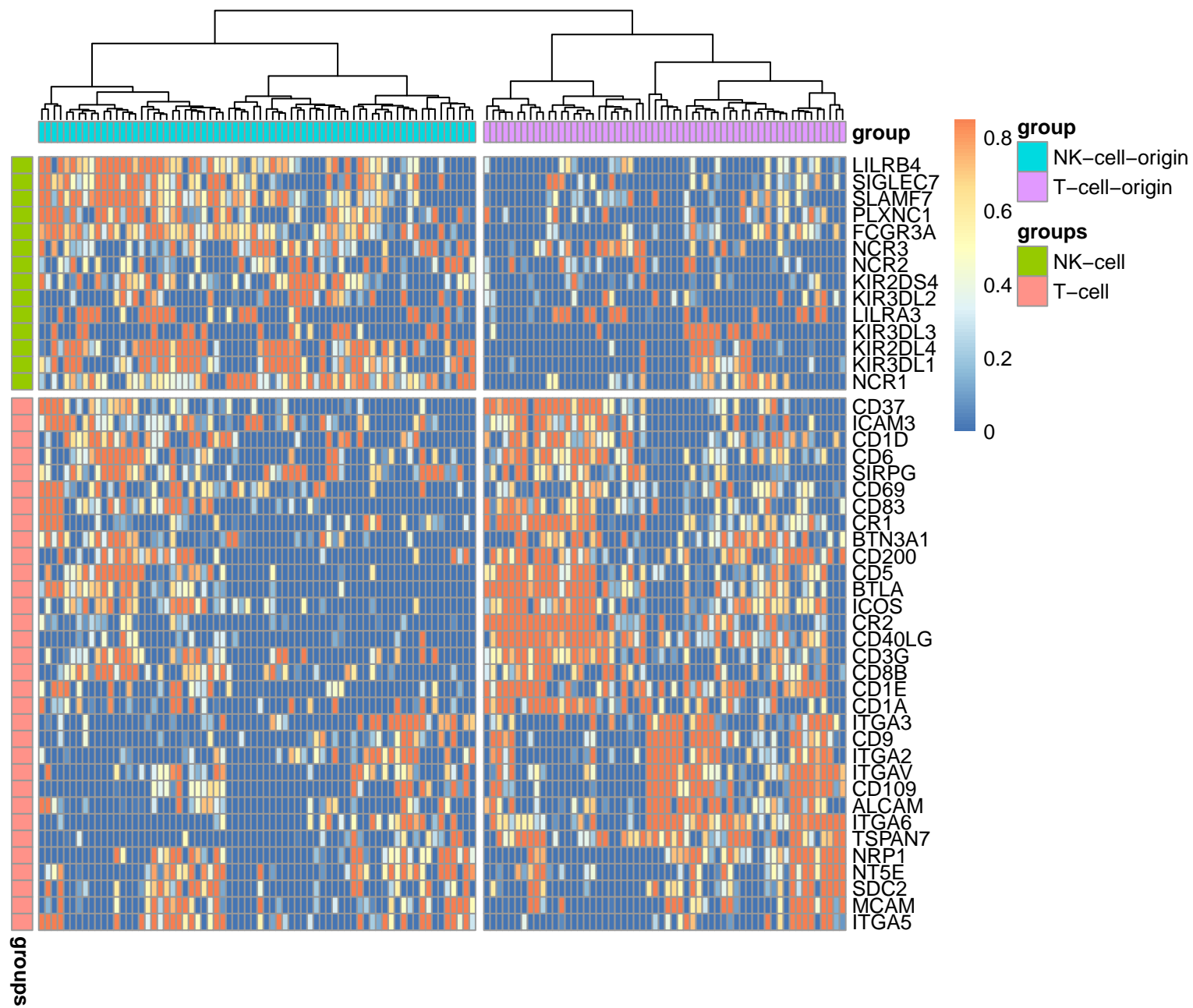

Supplement: Supplementary Figure 1 — Survival analysis according to the expression status of CD56 in patients with advanced stage NKTCL. (A) Overall survival (OS); (B) Progression-free survival (PFS). [file DataSheet_1.zip › supplemental Figure 2.pdf]

CD56

$3.2e-06$

value

15

10

5

NK-cell-origin

T-cell-origin

group

T-cell-origin  
NK-cell-origin

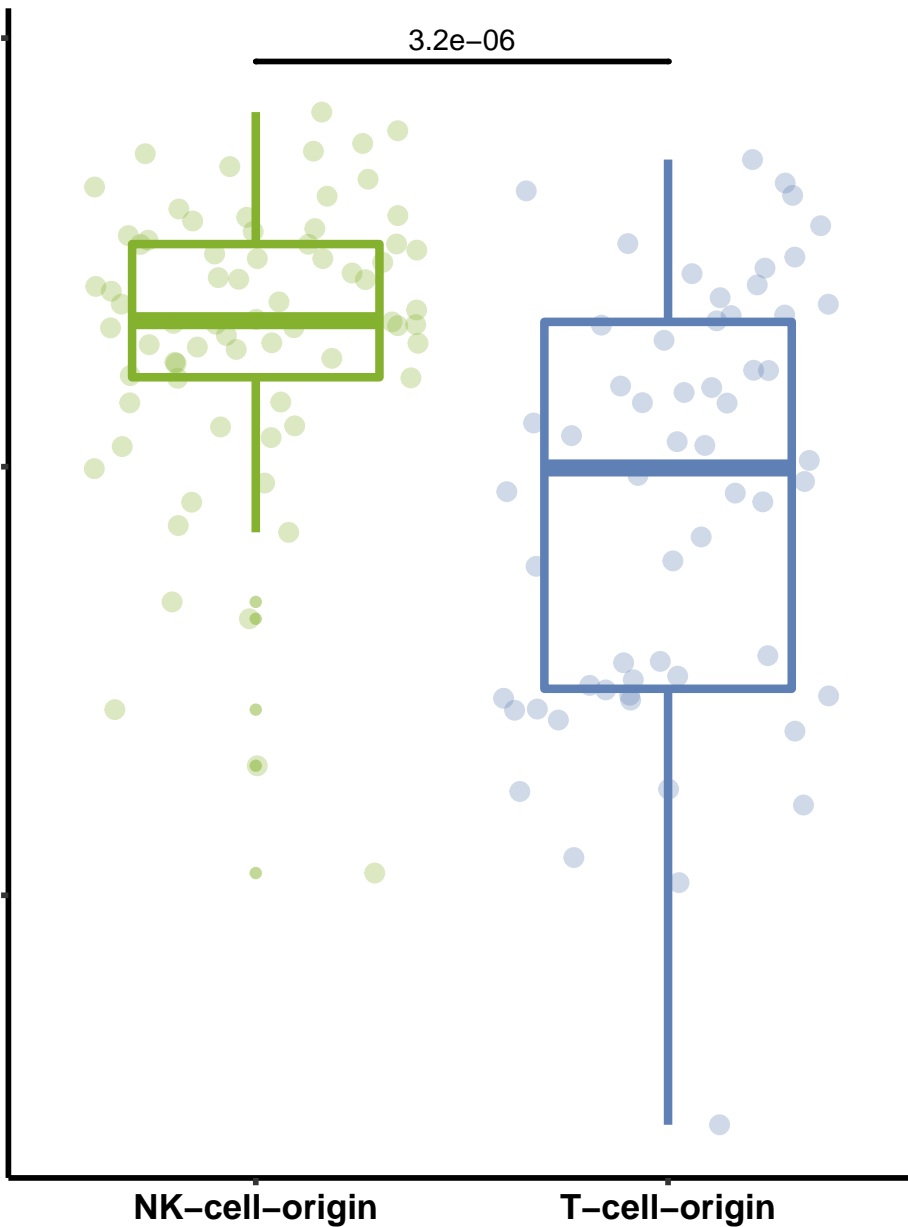

Supplement: Supplementary Figure 1 — Survival analysis according to the expression status of CD56 in patients with advanced stage NKTCL. (A) Overall survival (OS); (B) Progression-free survival (PFS). [file DataSheet_1.zip › supplemental Figure 3.pdf]

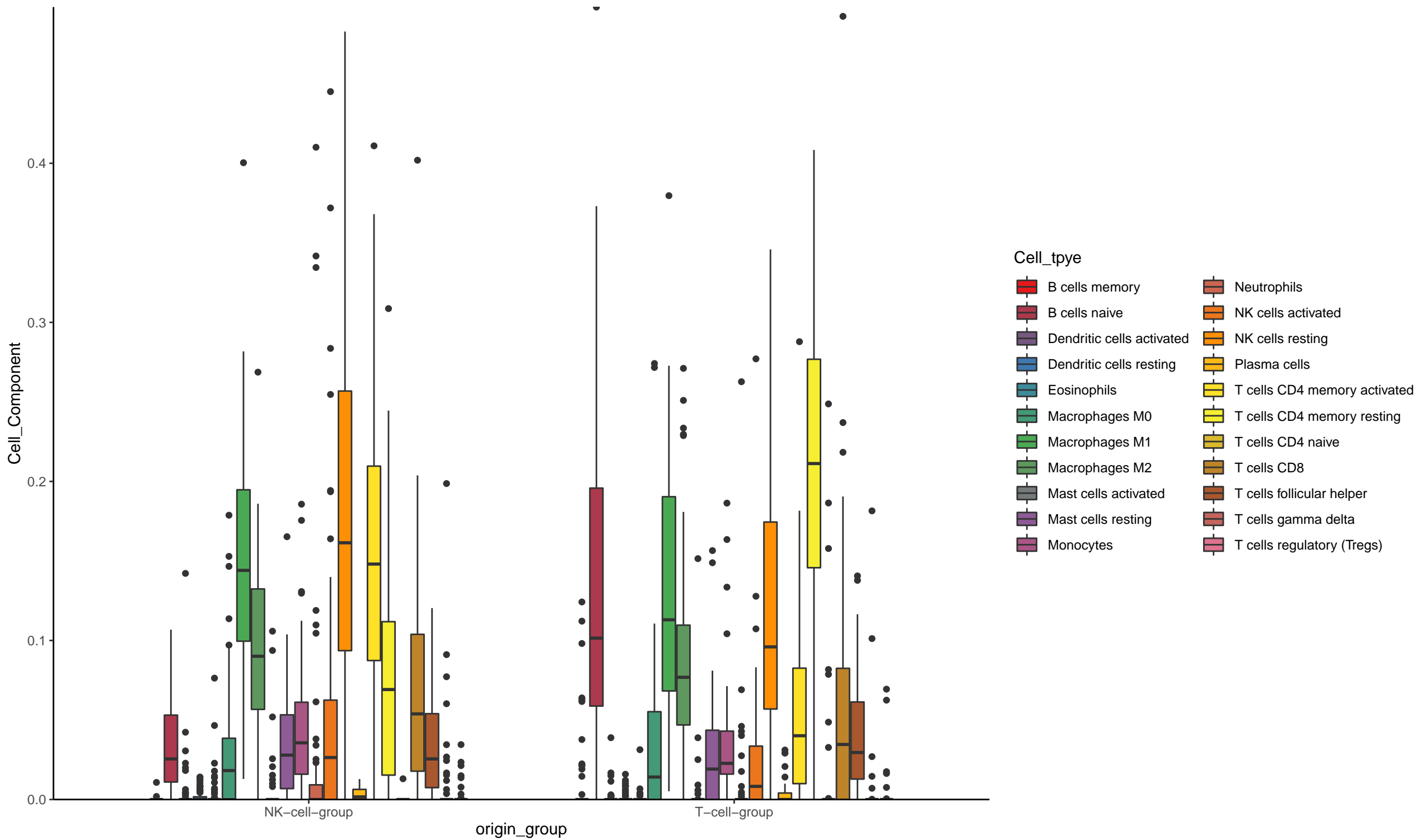

Supplement: Supplementary Figure 1 — Survival analysis according to the expression status of CD56 in patients with advanced stage NKTCL. (A) Overall survival (OS); (B) Progression-free survival (PFS). [file DataSheet_1.zip › supplemental Figure 4.pdf]

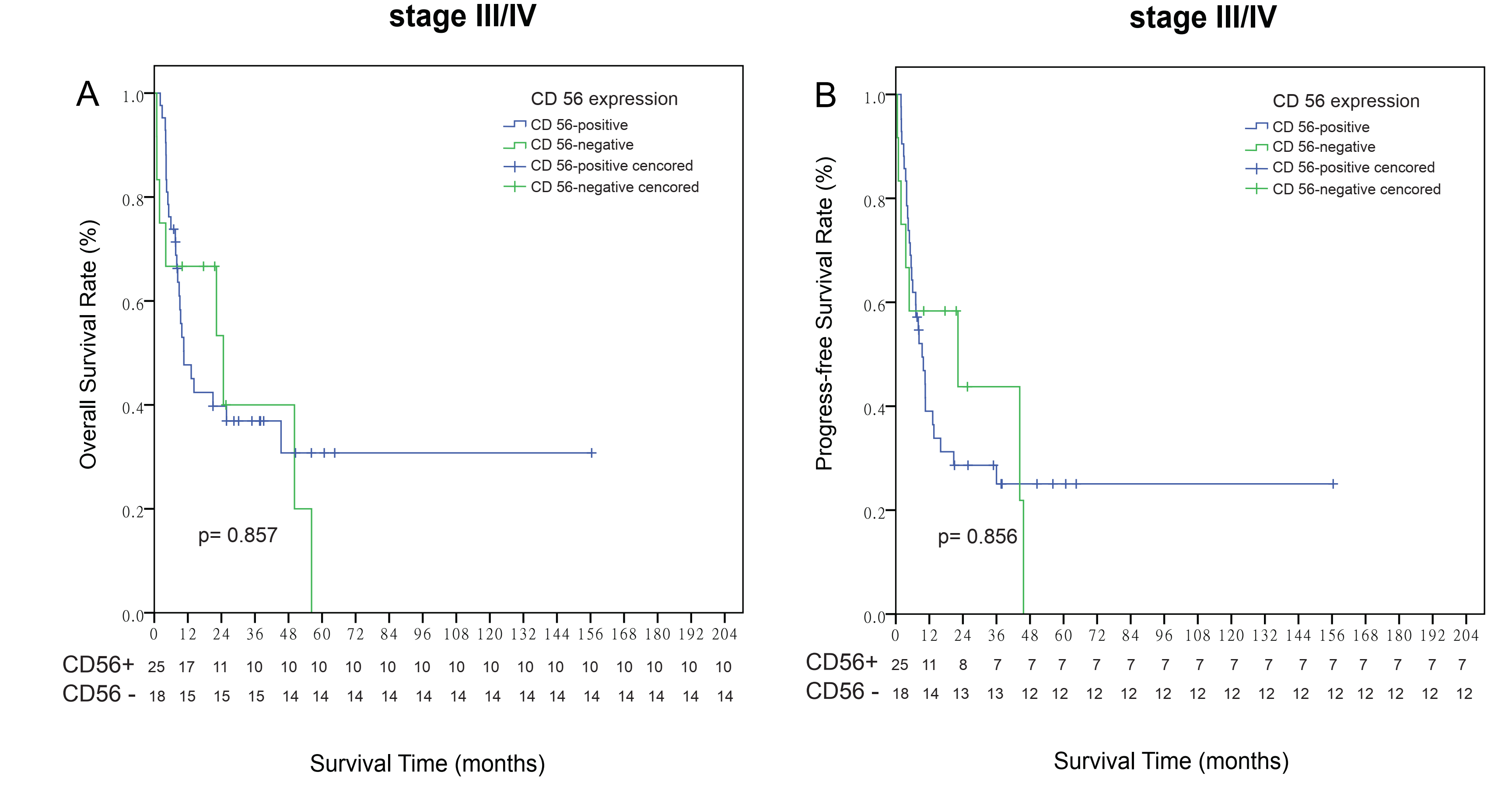

Supplement: Supplementary Figure 1 — Survival analysis according to the expression status of CD56 in patients with advanced stage NKTCL. (A) Overall survival (OS); (B) Progression-free survival (PFS). [file DataSheet_1.zip › supplemental Figure 1.tif]
